# Treatment for secondary hyperparathyroidism focusing on parathyroidectomy

**DOI:** 10.3389/fendo.2023.1169793

**Published:** 2023-04-20

**Authors:** Takahisa Hiramitsu, Yuki Hasegawa, Kenta Futamura, Manabu Okada, Norihiko Goto, Shunji Narumi, Yoshihiko Watarai, Yoshihiro Tominaga, Toshihiro Ichimori

**Affiliations:** Department of Transplant and Endocrine Surgery, Japanese Red Cross Aichi Medical Center Nagoya Daini Hospital, Nagoya, Japan

**Keywords:** secondary hyperparathyroidism, parathyroidectomy, chronic kidney disease, autotransplantation, mortality, calcimimetics

## Abstract

Secondary hyperparathyroidism (SHPT) is a major problem for patients with chronic kidney disease and can cause many complications, including osteodystrophy, fractures, and cardiovascular diseases. Treatment for SHPT has changed radically with the advent of calcimimetics; however, parathyroidectomy (PTx) remains one of the most important treatments. For successful PTx, removing all parathyroid glands (PTGs) without complications is essential to prevent persistent or recurrent SHPT. Preoperative imaging studies for the localization of PTGs, such as ultrasonography, computed tomography, and ^99m^Tc-Sestamibi scintigraphy, and intraoperative evaluation methods to confirm the removal of all PTGs, including, intraoperative intact parathyroid hormone monitoring and frozen section diagnosis, are useful. Functional and anatomical preservation of the recurrent laryngeal nerves can be confirmed *via* intraoperative nerve monitoring. Total or subtotal PTx with or without transcervical thymectomy and autotransplantation can also be performed. Appropriate operative methods for PTx should be selected according to the patients’ need for kidney transplantation. In the case of persistent or recurrent SHPT after the initial PTx, localization of the causative PTGs with autotransplantation is challenging as causative PTGs can exist in the neck, mediastinum, or autotransplanted areas. Additionally, the efficacy and cost-effectiveness of calcimimetics and PTx are increasingly being discussed. In this review, medical and surgical treatments for SHPT are described.

## 1 Introduction

During the early stages of chronic kidney disease (CKD), serum fibroblast growth factor-23 (FGF-23) increases to prevent hyperphosphatemia. However, FGF-23 can significantly suppress the synthesis of activated vitamin D, 1α,25-dihydroxyvitamin D. As the CKD progresses, further decreases in activated vitamin D and increases in the serum phosphorus levels can cause hypocalcemia. Additionally, the decreased expression of the vitamin D and calcium-sensing receptors expressed on the parathyroid cells can worsen secondary hyperparathyroidism (SHPT) (1). Severe SHPT can occur during long-term treatment for end-stage renal disease (ESRD). Furthermore, the incidence of severe SHPT with intact parathyroid hormone (PTH) levels >300 pg/mL is estimated to be approximately 33% (2).

For the treatment of SHPT, phosphorus binders and vitamin D receptor activators were developed; however, they were ineffective in cases of severe SHPT (1). For those with severe SHPT, the parathyroid glands (PTGs) progress to nodular hyperplasia, in which vitamin D receptor expression is decreased (3, 4). Although phosphorus binders are useful to improve serum phosphate levels, they do not reduce serum PTH effectively (5). Therefore, parathyroidectomy (PTx) is the radical surgical treatment for severe SHPT. PTx helps to improve bone mineral density, bone fracture risk, patient survival, and quality of life (QOL) (6–10).

In addition to the medical and surgical treatments, interventional treatment such as ultrasound-guided percutaneous ethanol injection (PEIT) is indicated for patients with only one enlarged PTG with a volume of >0.5 cm^3^ (11, 12). An advantage of interventional treatment is that it can be performed without general anesthesia and operation. In long-term ESRD conditions, patients with SHPT do not always tolerate general anesthesia and operation, and interventional regimens can therefore be a good treatment option (13–17). However, disadvantages include the risk of pain, hematoma, and recurrent laryngeal nerve (RLN) injury (12). The 66–85% success rate of PEIT is slightly lower than that of PTx, with persistence and recurrence rates of 5–30% (11, 18–22). Additionally, the number of patients indicated for interventional treatment is limited (11, 12). The mainstream SHPT treatment remains medical and surgical.

Recently, the development of calcimimetics has dramatically reduced the number of surgical treatments needed (23, 24). However, PTx for SHPT is still required considering its cost-effectiveness and application for drug-refractory SHPT (25–27). The difficulty of PTx for SHPT differs from PTx for primary hyperparathyroidism (PHPT) because complete removal of all PTGs is required to prevent persistent or recurrent SHPT (28). In PTx for PHPT, identification of PTGs is relatively easy (29), and causative PTGs are often identified during preoperative imaging studies (29). The Miami criteria for intraoperative intact parathyroid hormone (IOIPTH) monitoring have been established and are useful for confirming the resection of causative PTGs during PTx for PHPT (30). However, in PTx for SHPT, preoperative imaging studies can rarely identify all PTGs (20, 31), and criteria for IOIPTH monitoring have not been established. Additionally, PTGs firmly adhering to the RLNs are sometimes identified due to hemorrhage or calcification in the PTGs; thus, removing these PTGs may result in RLN injury (32).

When persistent or recurrent SHPT is suspected, localization of causative PTGs is required. However, when total PTx with autotransplantation is performed in the initial PTx, localization becomes particularly challenging as causative PTGs can occur in the autografted, cervical, or mediastinum areas (33). Additionally, the selection of timely and appropriate treatment requires an understanding of the differences between PTx and medical treatment with calcimimetics.

**Figure 1:**
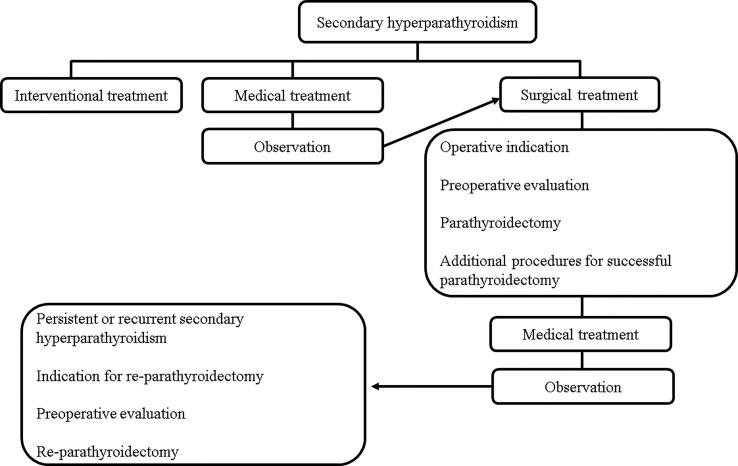
Flowchart of secondary hyperparathyroidism treatment.

**Figure 2:**
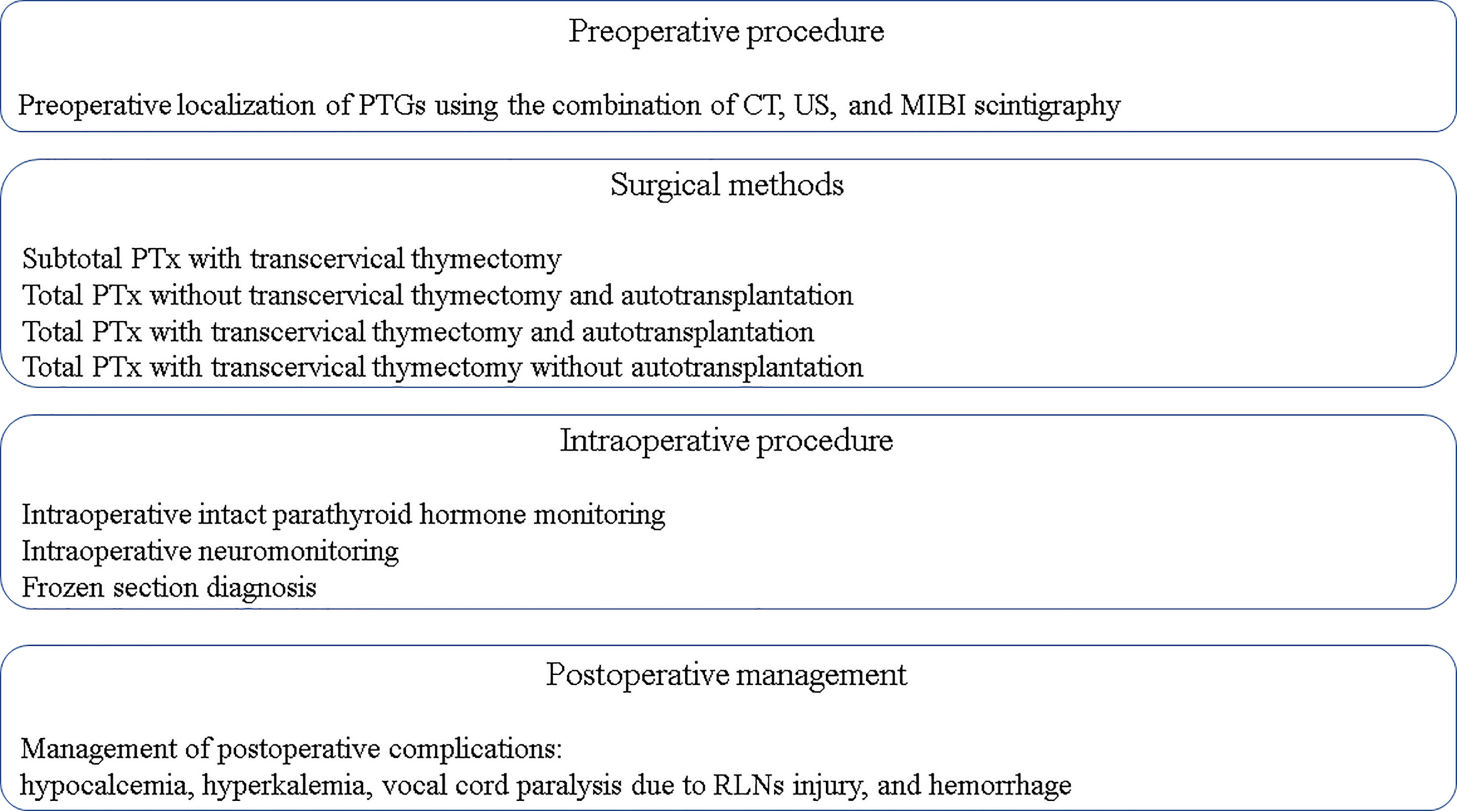
Key principles to achieve a successful PTx. CT, computed tomography; MIBI scintigraphy, ^99m^Tc-Sestamibi scintigraphy; PTx, parathyroidectomy; PTGs, parathyroid glands; RLNs, recurrent laryngeal nerves; US, ultrasonography.

In recent years, considering the popularity and availability of calcimimetics, the rate of PTx has decreased, and it has become difficult for young surgeons to gain experience performing PTx for SHPT (23, 24). However, PTx remains an important treatment option for SHPT. In this review, the medical and surgical treatments for SHPT were comprehensively explained to serve as a reference for the next generation of surgeons and to improve patient outcomes (**Figures 1**, **2**).

## 2 Indication for PTx

The Kidney Disease Improving Global Outcomes (KDIGO) 2009 and Japanese Society for Dialysis Therapy 2012 guidelines have defined the operative indications for SHPT (34, 35). In the KDIGO guidelines, the target range for intact PTH levels is within 2–9 times the upper normal range for patients with stage 5D CKD, equivalent to 130–600 pg/mL. When marked changes are identified in intact PTH levels, treatment initiation or a change in therapy is suggested. PTx is recommended when medical therapy has failed (34). In the Japanese Society for Dialysis Therapy 2012 guidelines, the target range for PTH is 60–240 pg/mL (35), and PTx is recommended when the intact PTH level is >500 pg/mL or whole PTH is >300 pg/mL (35, 36). Although the intact and whole PTH levels are lower than the suggested levels in hyperphosphatemia or hypercalcemia refractory to medical treatment, both conditions are indications for PTx. The target ranges for serum phosphorus and calcium are defined in the the National Kidney Foundation’s Kidney Disease Outcomes Quality Initiative (KDOQI) 2003 and Japanese Society for Dialysis Therapy 2012 guidelines (35, 37). Additionally, subjective complaints related to SHPT, including high bone turnover, significant changes on radiography, and ectopic calcification, can be positive indications for PTx (35). In the KDIGO 2017 clinical practice guideline update, medical therapy using calcimimetics, calcitriol, or vitamin D analogs are additionally suggested as PTH-lowering therapy (38). However, PTx is still regarded as a “valid treatment option” when medical therapy has failed, as suggested in the KDIGO 2009 guideline (34, 38).

## 3 Preoperative imaging evaluations for PTx in SHPT

During PTx for SHPT, preoperative imaging evaluations of the swollen PTGs are essential to ensure complete removal. In SHPT, CKD stimulates all PTGs, which should be removed in the initial PTx. However, remnant PTGs are also stimulated and cause persistent or recurrent SHPT (28, 39). Re-PTx for persistent or recurrent SHPT increases the risk of RLN injury owing to PTG adhesion from the initial PTx (40, 41). However, it is sometimes difficult to identify supernumerary and ectopic PTGs, and even locating the four main PTGs, namely the right upper and lower PTGs and left upper and lower PTGs, intraoperatively can be challenging (31, 42, 43). Supernumerary and ectopic PTGs are frequently located around the upper neck area, in the carotid sheath, within the thyroid gland, in the mediastinum, and in the thymus (18, 19, 31). Moreover, the color and shape of PTGs are similar to those of adipose tissues and lymph nodes. These similarities and unexpected locations often result in the misidentification of PTGs and, ultimately, unsuccessful PTx (44).

To minimize misidentification, preoperative imaging studies are essential, including ultrasonography (US), computed tomography (CT), and ^99m^Tc-Sestamibi (MIBI) scintigraphy (45). US is a cost-effective, non-invasive, and non-radiographic imaging test; however, it is difficult to identify ectopic PTGs in the mediastinum. Conversely, CT and MIBI scintigraphy are radiographic imaging tests. CT is useful to identify the four main and ectopic PTGs, although PTGs in the thymus and thyroid gland are rarely identified. MIBI scintigraphy is useful in identifying ectopic PTGs especially in the mediastinum and upper neck area, although diagnostic accuracy is low due to false positives and false negatives (20, 45). The diagnostic accuracy of these imaging studies has been investigated (20, 43, 46–49). US showed the highest sensitivity (91.5%) and MIBI scintigraphy had the lowest sensitivity (56.1%). The sensitivity of combined US and CT and combined US, CT, and MIBI scintigraphy had a sensitivity value of 95.0% and 95.4%, respectively (47). Additionally, in a meta-analysis, the sensitivity and specificity of the MIBI scintigraphy was 58% and 93%, respectively (48). However, evaluating the diagnostic accuracy is challenging because the definition of the removal of all PTGs is ambiguous in these studies. Moreover, the number of PTGs differs among patients (50).

In our previous study, the diagnostic accuracies of US, CT, and MIBI scintigraphy were investigated in patients with intact PTH levels of <9 pg/mL on postoperative day one, which was lower than the normal range and indicated the complete removal of the PTGs (20). The diagnostic accuracy for the usual four PTGs on US, CT, MIBI scintigraphy, combined US and CT, combined US and MIBI scintigraphy, combined CT and MIBI scintigraphy, and combined US, CT, and MIBI scintigraphy were 57.3%, 60.0%, 42.7%, 73.0%, 65.8%, 66.1%, and 75.1%, respectively. Combining US, CT, and MIBI scintigraphy had the highest accuracy for locating PTGs before PTx (20). Nevertheless, the diagnostic accuracy for locating the usual four PTGs was only 75.1% because approximately 18% (247 out of 1332 PTGs) were ectopic. Additionally, approximately 90% (231 out of 247 PTGs) of these ectopic PTGs are located in the thymus, making identification *via* imaging modalities challenging, considering the low sensitivity of MIBI scintigraphy and that the CT density and US pattern of PTGs is similar to those of lymph nodes and adipose tissues (31). The diagnostic accuracy for ectopic PTGs on US, CT, MIBI scintigraphy, combined US and CT, combined US and MIBI scintigraphy, combined CT and MIBI scintigraphy, and combined US, CT, and MIBI scintigraphy were 36.7%, 47.8%, 30.0%, 60.0%, 48.9%, 57.8%, and 63.3%, respectively (31). While these results for ectopic PTGs were similar to the four main PTGs, the diagnostic accuracy for ectopic PTGs was somewhat lower. This implies that the identification of ectopic PTGs *via* preoperative imaging is more difficult than that of the usual PTGs (31).

Supernumerary PTGs also increase the difficulty of PTx. Approximately 6% of all resected PTGs (44 out of 747 PTGs or 90 out of 1422 PTGs) were supernumerary PTGs. None of the supernumerary PTGs were identified by US, CT, and MIBI scintigraphy. Notably, around 70% (64 out of 90 PTGs) were too small to be identified intraoperatively. These very small PTGs are referred to as micro nests and can only be identified by paraffin section diagnosis (31).

Persistent or recurrent SHPT can occur in 5–30% of patients, and re-PTx is required (18–21). The diagnostic accuracy of re-PTx has been investigated in a limited number of patients. The diagnostic accuracies of CT and MIBI scintigraphy were both 100%, although that of US was only 28% (31). Causative PTGs of persistent or recurrent SHPT are often located in the thymus and mediastinum; however, US cannot detect these because of the sternum and clavicle bones. In cases of re-PTx, preoperative CT and MIBI scintigraphy are thus required to localize the causative PTGs.

For the initial PTx, the accuracy of each imaging modality is not high. Therefore, for the precise preoperative localization of PTGs, combining US, CT, and MIBI scintigraphy is the favorable option where appropriate. Additionally, for re-PTx in the thymus and mediastinum, CT and MIBI scintigraphy are useful.

Recently, single photon emission computed tomography (SPECT/CT) has also been utilized for preoperative imaging evaluations. SPECT/CT is the fusion of CT and MIBI scintigraphy, which increases the localization accuracy of MIBI scintigraphy (43, 51, 52). For preoperative localization of PTGs, the findings of CT and US should be carefully evaluated prior to PTx.

## 4 Surgical procedure of PTx for SHPT

The surgical procedure can consist of a combination of total or subtotal PTx, transcervical thymectomy, and autograft. The following four procedures are often described in reports:

1. Subtotal PTx with transcervical thymectomy
2. Total PTx without transcervical thymectomy and autotransplantation
3. Total PTx with transcervical thymectomy and autotransplantation
4. Total PTx with transcervical thymectomy and without autotransplantation

In this section, the advantages and disadvantages of these surgical procedures are compared and reviewed. First, the frequency of these four procedures in previous reports was analyzed. Total PTx with transcervical thymectomy and autograft is the most common globally at 68.1%, followed by subtotal PTx with transcervical thymectomy at 19.8%, total PTx without transcervical thymectomy and autograft at 10.3%, and total PTx with transcervical thymectomy and without autograft at 1.6% (53).

Subtotal PTx with transcervical thymectomy involves removing 3.5 PTGs and leaving 40–80 mg of PTG tissue. PTGs with a normal appearance are favored for preservation to avoid recurrence. Transcervical thymectomy is usually added to prevent persistent and recurrent SHPT due to ectopic or supernumerary PTGs in the thymus. The frequency of ectopic and supernumerary PTGs in the thymus is 22.0–39.3% and 6.5–37%, respectively (19, 20, 49, 50, 54). The advantage of this procedure is that hypoparathyroidism can be avoided by leaving a PTG. However, there is a potential risk of direct dissemination to the surroundings and hematogenous dissemination to other organs from the PTG stump (55). Persistent or recurrent SHPT due to dissemination makes it almost impossible to remove causative disseminated PTGs (55), whereas persistent and recurrent SHPT due to the remaining PTG requires re-PTx.

Total PTx without transcervical thymectomy and autograft is performed to prevent hypoparathyroidism after PTx as PTH secretion is expected from the ectopic and supernumerary PTGs in the thymus. One disadvantage is the potential risk of recurrent or persistent SHPT due to the remnant PTGs in the thymus, which increases the risk of RLN injury in the case of re-PTx (56). The causative PTGs in the thymus in persistent or recurrent SHPT are comparatively close to the RLNs. For safe re-PTx, intraoperative neuromonitoring (IONM) is recommended because of adhesion due to the initial PTx (57). Another disadvantage is that general anesthesia is essential in the case of re-PTx. Patients with severe SHPT often experience other complications such as cardiovascular diseases and cervical destructive spondyloarthropathy owing to long-term CKD (13–17). These complications can cause unstable vital signs during general anesthesia and cervical spine injury during intubation or surgical positioning. Furthermore, there is a potential risk of postoperative bleeding as a result of the dissection of adhesions and fragile tissues associated with CKD. Postoperative bleeding can narrow the trachea and potentiate the risk of suffocation. Therefore, re-PTx should be avoided in patients with SHPT. However, subtotal PTx with transcervical thymectomy and total PTx without transcervical thymectomy and autograft are selected for patients expecting kidney transplantation in the near future as improved kidney function after transplantation does not stimulate the remnant PTGs; therefore, persistent or recurrent SHPT can be avoided (58). Additionally, although improved kidney function can cause hypoparathyroidism, the PTH secreted from remnant PTGs can avoid this problem (59).

Total PTx with transcervical thymectomy and autograft is the most common procedure globally (53). The risk of persistent or recurrent SHPT in the neck can be almost entirely prevented after successful PTx. Moreover, hypoparathyroidism is prevented by the expected secretion of PTH from the autograft (60). This procedure is favored in both patients expecting long-term dialysis and those expecting kidney transplantation (61). The most common location for the autograft is the forearm since removal of the recurrent PTGs can easily be performed under local anesthesia. However, the diagnosis of causative PTGs in persistent or recurrent SHPT is complicated. The neck and mediastinum areas and forearm are distinct locations which cannot be examined simultaneously. Methods to locate causative PTGs are reviewed in Section 6.

Total PTx with transcervical thymectomy without autograft is rarely performed due to the complete lack of PTH secretion (53). The impact of hypoparathyroidism after PTx has also not been fully revealed (62). In the KDIGO 2009 guidelines, this procedure was contraindicated for patients expecting kidney transplantation because hypoparathyroidism after transplantation may cause serious bone metabolism problems and hypocalcemia (34).

## 5 Comparison of PTx procedures

### 5.1 Subtotal PTx versus total PTx with autotransplantation

Literature comparing surgical procedures for SHPT is limited. Complications, reoperations, readmission, and 30-day mortality were similar between subtotal and total PTx with autotransplantation in a report based on data from the American Surgical College of Surgeons National Surgical Quality Improving Program (63). This study demonstrated similar short-term outcomes for both PTx procedures. Additionally, in a randomized trial, significant improvements in clinical signs and calcium levels were observed, and re-PTx was not identified in the total PTx with autotransplantation group. This study demonstrated the superiority of total PTx with autotransplantation compared to subtotal PTx (64).

In a retrospective study by the Swedish Renal Registry, long-term outcomes were investigated (65), and the risk of cardiovascular events was lower in subtotal than in total PTx. This can be explained by the lower PTH levels in total than in subtotal PTx, although 78% of patients underwent autotransplantation after total PTx. Low bone turnover due to low PTH levels can increase the calcification of coronary arteries (66, 67). The re-PTx rate is higher in subtotal than in total PTx due to the low kidney transplantation rate in this group (65), although previous reports demonstrated similar re-PTx rates for subtotal and total PTx (68). Rates of hip fracture, paralysis of RLNs, and mortality are similar between subtotal and total PTx (65). Furthermore, in a systematic review on the prevention of SHPT after subtotal or total PTx with autotransplantation, symptomatic improvement, radiological change, hypocalcemia rate, persistence rate, time to recurrence, recurrence rate, and reoperation rates were similar (69).

The QOL between subtotal and total PTX with bilateral cervical thymectomy and autotransplantation was investigated using the 36-item Short Form Health Survey questionnaire (6). In this prospective randomized trial, there were no significant differences in QOL between procedures for SPHT. The study concluded that PTx can improve the QOL of patients with SHPT regardless of the surgical procedure.

These studies imply that total PTx is favored for patients who need long-term dialysis, although special attention is necessary for cardiovascular events. However, subtotal PTx can be indicated for patients with SHPT expecting kidney transplantation in the near future (70, 71).

### 5.2 Total PTx without autotransplantation versus total PTx with autotransplantation and transcervical thymectomy

In most of the literature regarding total PTx with autotransplantation, transcervical thymectomy was performed. Regarding the short-term outcomes investigated in a multicenter prospective randomized controlled pilot trial, paralysis of RLNs, postoperative bleeding, and postoperative hypocalcemia were similar in both total PTx without autotransplantation and total PTx with autotransplantation and transcervical thymectomy. Moreover, mortality after 3 years of follow-up was also similar (72). However, PTH levels were significantly higher at the end of follow-up after PTx with autotransplantation and transcervical thymectomy.

In a systematic review involving 1108 patients across 11 studies, symptomatic improvement, surgical complications such as postoperative bleeding, RLN paralysis, and all-cause mortality were similar for both procedures. Re-PTx owing to persistent or recurrent SHPT was performed more frequently in total PTx with autotransplantation and transcervical thymectomy (73). Hypocalcemia was more frequently identified in total PTx without autotransplantation, although hypoparathyroidism was similar between the two surgical procedures. Similarly, in a meta-analysis including 1283 patients across 10 studies, persistent or recurrent SHPT occurred more frequently after total PTx with autotransplantation and transcervical thymectomy. However, hypoparathyroidism often develops after total PTx without autotransplantation, although permanent hypocalcemia and adynamic bone disease were not reported (60). These studies demonstrated similar short- and long-term outcomes for both procedures, except for the higher rate of re-PTx for persistent or recurrent SHPT in total PTx with autotransplantation and transcervical thymectomy.

It should be noted that in these studies the locations of causative PTGs were not revealed in total PTx with autotransplantation and transcervical thymectomy. If causative PTGs in the neck or mediastinum can be identified more frequently in total PTx with autotransplantation and transcervical thymectomy, the success rate of PTx should be improved, reducing persistent or recurrent SHPT. In this case, the impact of both procedures on persistent or recurrent SHPT cannot be compared. When initial PTx is successful, persistent or recurrent SHPT is caused by the transplanted autograft and appropriate PTGs for the autograft should be discussed in more detail. PTGs with nodular hyperplasia more frequently cause recurrent SHPT than diffuse glands (74). Previous reports suggested that PTGs with a maximum diameter >8 mm on preoperative US can indicate nodular hyperplastic changes (75). This implies that PTGs for autografts should be fragmented from PTGs with a maximum diameter <8 mm on preoperative US. However, the intraoperative characteristics of appropriate PTGs to prevent recurrent SHPT have not been investigated, although the relationships between intraoperative parameters and pathological patterns have been reported (76, 77).

### 5.3 Subtotal PTx versus total PTx without autotransplantation versus total PTx with autotransplantation and transcervical thymectomy

Only one systematic review comparing the above three surgical procedures was identified (78). This systematic review included 5063 patients across 26 reports (78). Hypocalcemia and hypoparathyroidism were most frequently identified in total PTx without autotransplantation, whereas the frequency was similar in subtotal PTx and total PTx with autotransplantation and transcervical thymectomy. Persistent or recurrent SHPT and re-PTx were most common in subtotal PTx. Based on these results, total PTx with autotransplantation and transcervical thymectomy was recommended for SHPT.

This comparison implies that subtotal PTx is not appropriate for patients who require long-term dialysis owing to the high incidence of persistent or recurrent SHPT caused by the remaining PTGs. In contrast, patients who require kidney transplantation can be indicated for subtotal PTx. However, the impact of hypoparathyroidism after total PTx without autotransplantation on bone metabolism, cardiovascular events, and mortality in the long term (e.g., 10 years) has not been reported. Patients who undergo total PTx without autotransplantation may experience hypoparathyroidism and hypocalcemia after kidney transplantation. Thus, avoiding total PTx without autotransplantation is preferred for optimal patient outcomes.

At present, total PTx with autotransplantation and transcervical thymectomy is the best option for SHPT. The rate of persistent or recurrent SHPT originating from PTGs in the neck or mediastinum area is low. Recurrent SHPT caused by the autograft can easily be addressed by the removal of the causative PTGs under local anesthesia. The appropriate PTx procedure should be selected based on the patient’s condition and the schedule for kidney transplantation.

## 6 Additional methods during PTx

Additional measurements to ensure successful PTx, including IONM, IOIPTH monitoring, frozen section diagnosis, and other methods are reviewed in this section.

### 6.1 IONM

Proper identification of PTGs and the presence of RLNs contribute to the difficulty of PTx. Identification of RLNs is essential during PTx to prevent RLN injury, which can lead to hoarseness and difficulty in swallowing and breathing. IONM was developed to prevent RLN injuries. Electronic stimulation of the RLNs causes movements of the vocal cord, detected by the attachment around the tracheal tube (79). This response to IONM implies anatomical and functional preservation of the RLNs.

The diagnostic accuracy of IONM in the initial PTx is 94.7%, suggesting that a positive or negative IONM response during PTx can predict intact vocal cord movement or vocal cord paralysis in 94.7% of RLNs (32). Although the diagnostic accuracy of IONM during PTx is high, preserving the RLNs is difficult in some cases. Inflammation due to calcification and hemorrhage of the PTGs can cause severe adhesion of the PTGs to the RLNs (80). In these cases, accurate dissection is required, and IONM should be indicated. In cases with severe adhesion of the PTGs to the RLNs, IONM allows the incidence of vocal cord paralysis to be lowered to a similar incidence as without severe adhesion (32). Predictive factors for severe adhesion of the PTGs to the RLNs are a maximum diameter >15 mm, weight >500 mg, and nodular hyperplasia. Based on these parameters, IONM should be indicated when PTGs with a maximum diameter >15 mm are identified on preoperative imaging.

Although IONM is a useful method in PTx, the accuracy is not 100%. This might be due to false-positive cases in which a positive IONM response did not lead to intact vocal cord movement, possibly caused by delayed neuropraxia due to edema or laryngeal edema. Additionally, false-negative cases where intact vocal cord movement was identified despite a negative IONM response may be caused by malpositioning of the tracheal tube or temporary intraoperative RLN paralysis (79). These issues should be considered when using IONM.

### 6.2 IOIPTH monitoring

Intraoperative PTH monitoring was first developed to ensure the removal of causative PTGs in PHPT (30, 81–83). Preoperative PTH levels and PTH levels after the removal of causative PTGs are measured to investigate the appropriate decrease in PTH levels for predicting successful PTx. In PTx for PHPT, a 50% decrease 10 minutes after the removal of causative PTGs was demonstrated as the best indicator of successful PTx (30). Similarly, researchers have tried to adapt intraoperative PTH monitoring for PTx for SHPT. However, the condition of patients with SHPT is different from that of patients with PHPT. The kidney function of most patients with PHPT is within the normal range, whereas patients with SHPT have ESRD, which makes it difficult to establish criteria for intraoperative PTH monitoring.

PTH is a single-chain polypeptide hormone consisting of 84 amino acids (84). Whole PTH, referred to as 1-84 PTH, is biologically active and metabolized into multiple fragments in the liver and kidneys (85). Although whole PTH should be measured during intraoperative PTH monitoring, the widely available second-generation intact PTH assay kit that uses an enzyme-linked immunosorbent assay with a one-step sandwich method is problematic as cross-reactivity between 7-84 PTH and whole PTH causes a discrepancy between measured intact PTH and whole PTH levels, especially in patients with ESRD (86, 87). In healthy populations, the 7-84 PTH fragment is mainly excreted in the urine, and the impact of cross-reactivity is low. In patients with ESRD, the pharmacological half-life of 7-84 PTH is several hours owing to poor kidney function, whereas the pharmacological half-life of whole PTH is approximately 3–4 minutes. Although intraoperative PTH monitoring is expected to obtain results within 10–20 minutes during the operation, the discrepancy of the half-lives and cross-reactivity between 7-84 PTH and whole PTH make it difficult to establish the criteria for IOIPTH monitoring to predict successful PTx.

Additionally, establishment of IOIPTH monitoring criteria for the prediction of successful PTx requires reputable operative methods and techniques. In recent studies, these criteria were investigated (31, 88, 89). We previously showed that a 70% decrease in intact PTH 10 minutes after total PTx and transcervical thymectomy can predict successful PTx, defined as an intact PTH level <60 pg/mL on postoperative day one (88). This was considered to be the definition of successful PTx because the incidence of re-PTx for recurrent or persistent SHPT was significantly lower in patients with an intact PTH level <60 pg/mL on postoperative day one (31, 88). The accuracy of this criterion was 92.9%. Among 226 patients, 26 benefited from IOIPTH monitoring. In five patients, IOIPTH did not decrease >70%, and further exploration was needed to remove the additional PTGs. In 21 patients, although fewer than four PTGs were removed, IOIPTH decreased by >70%, indicating that the operation was complete, avoiding potential RLN injury. The limitation of this criterion is its low specificity (52.2%). This is caused by patients in whom IOIPTH decreased by >70% but intact PTH on postoperative day one was >60 pg/mL, indicating the existence of residual PTGs despite the decrease in IOIPTH. Subsequently, these criteria were further investigated by Zhang et al. (89). Total PTx without thymectomy was performed, and the definition of successful PTx was intact PTH <50 pg/mL 1 week after PTx. The criterion for successful PTx was a >88.9% decrease in IOIPTH 20 minutes after total PTx without thymectomy, with a positive predictive value of 97.1% and a negative predictive value of 26.5%.

Following these studies, despite the different surgical procedures and definitions of successful PTx, the appropriate criteria for IOIPTH monitoring during PTx for SHPT were established. These IOIPTH criteria may help ensure successful PTx. Recently, a third-generation PTH assay has become available (90), in which whole PTH can be measured without cross-reactivity with 7-84 PTH. However, as intraoperative PTH monitoring during PTx for SHPT has not been investigated using this assay, appropriate criteria for successful PTx remain to be elucidated.

### 6.3 Frozen section diagnosis

The importance of frozen section diagnosis has rarely been investigated (31); however, the significance might depend on the availability of pathologists. In our previous study, IOIPTH monitoring and frozen section diagnosis were independent contributing factors to successful PTx (31, 91). The importance of IOIPTH is discussed in Section 6.2. In terms of frozen section diagnosis, the number of PTGs identified was a significant contributing factor according to the multivariate logistic regression analysis (31). Additionally, the diagnostic accuracies of frozen section and surgeons for PTGs were investigated by comparison with paraffin section diagnosis. Frozen section diagnosis had a higher diagnostic accuracy than surgeons at 99.4% and 88.9%, respectively. This implies that the diagnosis of PTGs by surgeons is not sufficient. Thyroid glands, lymph nodes, and adipose tissue were misdiagnosed by surgeons as PTGs, and the causes of these misdiagnoses were investigated. Notably, PTGs mimic the appearance of surrounding tissues, making it difficult for surgeons to distinguish them from the surrounding tissues. Moreover, although misdiagnosis using frozen sections is rare, thyroid glands and lymph nodes were misdiagnosed as PTGs. As mentioned in this study, using IOIPTH monitoring simultaneously improves the success of PTx as 6.6% of all resected PTGs, consisting of 4.9% supernumerary PTGs and 1.7% usual four PTGs, were not identified by frozen section diagnosis (31). To confirm the resection of these PTGs, IOIPTH monitoring is useful.

Additionally, in cases of total PTx and autograft for SHPT, the selection of PTGs for autograft is important. To avoid hypoparathyroidism and autografting lymph nodes or thyroid tissues involved in thyroid cancer, frozen section diagnosis is required. However, frozen section diagnosis should be performed by experienced pathologists. When PTx for SHPT is required and experienced pathologists are not available, patients should be referred to hospitals with experienced surgeons and pathologists.

### 6.4 Other methods

Radio-guided PTx is reported to be useful in identifying PTGs intraoperatively. In radio-guided PTx, preoperative MIBI is administered intravenously, and MIBI-accumulated PTGs are detected by the gamma counter. Radio-guided PTx for SHPT has been reported to be useful in identifying ectopic and undetected PTGs (92).

Recently, near-infrared autofluorescence imaging has been developed to identify PTGs without using a radio-active substance. Instead, this method uses the unique autofluorescent signature of PTGs under near-infrared wavelengths (93). The usefulness in clinical practice has been demonstrated (94–96). Currently, the only method to confirm the complete removal of PTGs during surgery is IOIPTH monitoring; however, near-infrared autofluorescence imaging has the potential to locate ectopic PTGs and those with a similar appearance to surrounding tissues. Nevertheless, the suitability of near-infrared autofluorescence imaging in PTx for SHPT remains to be investigated in large-scale studies (97–99).

Frozen section diagnosis is the most reliable method for confirmation of PTGs during surgery. However, parathyroid aspiration in which PTH levels in the resected PTGs are measured and used for the confirmation of PTGs is an alternative method with high sensitivity (100–102). The efficacy and cost-effectiveness of parathyroid aspiration should be further investigated with large trials. However, considering the cost-effectiveness, frozen section diagnosis may be replaced with parathyroid aspiration in the future after establishing its efficacy.

Overall, these additional methods, along with IONM, IOIPTH monitoring, and frozen section diagnosis, may be advantageous for PTx for SHPT. In particular, IONM is an indispensable method in thyroid and parathyroid surgeries as RLN injury can significantly deteriorate patient QOL. Further research with large-scale randomized trials is needed to fully elucidate the risks and benefits of each of these modalities, alone and in combination, for PTx in SHPT.

## 7 Postoperative complications and treatments

Postoperative complications include hypocalcemia, hyperkalemia, vocal cord paralysis due to RLNs injury, and hemorrhage. Following the significant decrease in PTH after PTx, marked skeletal uptake of calcium occurs and leads to hypocalcemia, named “hungry bone syndrome,” in which prominent bones form after PTx (103). Hypocalcemia is identified in 27% of patients on dialysis (61). Hypocalcemia can cause symptoms such as muscle cramps, tingling in the lips, tongue, fingers, and feet, and positive Chvostek or Trousseau sign. To prevent hypocalcemia, calcium replacement therapy is essential after PTx to maintain adequate serum calcium levels (37, 104, 105). After PTx for SHPT, “the blood level of ionized calcium should be measured every 4 to 6 hours for the first 48 to 72 hours after surgery and then twice daily until stable” according to KDOQI clinical practice guidelines for bone metabolism and disease in CKD (37).

Additionally, severe hyperkalemia is identified in 6.3% of patients after PTx for SHPT in a report of 1500 cases (106). In the case of postoperative severe hyperkalemia, urgent dialysis is required to prevent cardiovascular events. The risk factors of hyperkalemia after PTx for SHPT are not well demonstrated, although the relationships of hyperkalemia with gender, age, obesity, and preoperative serum potassium levels are mentioned in small-sized studies (107–109).

Postoperative hemorrhage is a serious complication leading to suffocation. Meticulous hemostasis is essential in preventing postoperative hemorrhage; however, postoperative hemorrhage can occur in 0.07–5% patients after thyroid and parathyroid surgery (110, 111). To reduce the compression to the trachea in case of postoperative hemorrhage, opening the wound without delay is the first and most important procedure. After securing the airway by endotracheal intubation or tracheostomy, reoperation under general anesthesia should be considered (110, 111).

Vocal cord paralysis due to RLNs injury is also a serious complication. In PTx for SHPT, RLN injury was identified in 2.47% cases in a report of 1500 cases (106). The prevention of RLN injury using IONM is essential, as discussed in Section 6.1.

## 8 Persistent or recurrent SHPT after PTx

### 8.1 Diagnosis of persistent or recurrent SHPT

The management of SHPT after PTx is not meticulously detailed in the clinical guidelines. For the follow-up of parathyroid function, serum PTH levels should be measured every 3 months in CKD stage 5 patients as mentioned in the KDOQI clinical practice guidelines for bone metabolism and disease in CKD (37). Similarly, persistent or recurrent SHPT has also not been defined in the guidelines. In previous reports, the definition of persistent or recurrent SHPT is usually based on the target serum intact PTH levels in the KDIGO guidelines 2009 or Japanese Society for Dialysis Therapy 2012 guidelines, with a target range of 2–9 times the upper normal range, equivalent to 130–600 pg/mL or 60–240 pg/mL, respectively (34, 35). Persistent SHPT is defined in some reports as serum intact PTH levels that do not decrease beyond the lower limit of the target range after PTx (28, 31, 33, 39, 88). In contrast, serum intact PTH level increasing above the upper limit should be defined as recurrent SHPT. However, the operative indication of initial PTx for SHPT with serum intact PTH levels >800 pg/mL in the KDOQI clinical practice guidelines for bone metabolism and disease in CKD or >500 pg/mL in Japanese Society for Dialysis Therapy 2012 guidelines is used for indication of re-PTx for persistent or recurrent SHPT (35, 37). Moreover, these intact PTH levels are used as a definition of recurrent SHPT in some reports (31, 88, 112).

Persistent or recurrent SHPT may stem from the neck, mediastinum, or autografted forearm (33). In total PTx, the incidence of persistent or recurrent SHPT in the cervical area or mediastinum is 5–30% (18–21). The incidence of recurrent SHPT in the autografted forearm is 5%, although the number of related reports is limited (113). Persistent or recurrent SHPT more frequently requires re-PTx instead of medical treatment as most cases are refractory to medical treatment (28, 37). Before re-PTx, the preoperative localization of causative PTGs is essential. In the case of total or subtotal PTx without autograft, only the neck and mediastinum need to be investigated for causative PTGs. In contrast, when autograft is performed, localization of causative PTGs is more complicated as both the neck or mediastinum and the autografted forearm are potential sites.

Although localization is performed using US, CT, MIBI scintigraphy, or magnetic resonance imaging, it is not cost-effective to simultaneously evaluate both the neck or mediastinum and the autografted forearm. Before performing these imaging studies, we need to infer the approximate site of the causative PTGs. The Casanova test or modified Casanova test was developed for this purpose (114, 115). By measuring the blood levels of PTH from the bilateral forearm and the autografted arm and comparing the ratio, localization of causative PTGs can be inferred. However, the number of enrolled patients in these studies was limited, and avascularization with a tourniquet or Esmarch bandage for >10 minutes is required. Thus, we developed a new criterion to reduce the burden on patients (33); the duration of avascularization was only 5 minutes to determine the cutoff ratio of intact PTH levels for the diagnosis of causative PTGs. The intact PTH ratio (intact PTH level obtained from the non-autografted forearm/intact PTH level obtained from the autografted forearm) was significantly lower in patients with causative PTGs in the autografted forearm than in those in the neck or mediastinum. Furthermore, receiver operating characteristic curve analyses revealed the appropriate cutoff ratio for localization. An intact PTH ratio <0.310 indicated localization in the autografted forearm; an intact PTH ratio >0.859 indicated localization in the neck or mediastinum. However, an intact PTH ratio between 0.310 and 0.859 indicated that both areas need to be examined. Using this algorithm for the localization of causative PTGs can facilitate the diagnosis and decrease procedural costs.

In addition to conventional methods like palpation, magnetic resonance imaging is useful for the diagnosis of causative PTGs in the forearm (116). For diagnosis in the neck or mediastinum, the diagnostic accuracy of US is reportedly only 28.6% compared with 100% for CT and MIBI scintigraphy (20). However, it should be noted that in that study 57.1% (4 out of 7) of causative PTGs were in the intrathymic lesion or mediastinum, and US could not detect these PTGs owing to the clavicles or sternum. Recently, SPECT/CT imaging has been introduced to replace CT and MIBI scintigraphy for the detection of PTGs. Nonetheless, the usefulness of SPECT/CT imaging for the diagnosis of persistent or recurrent SHPT still needs to be investigated (117).

Although postoperative management and operative indications of persistent or recurrent SHPT after PTx is not currently established in any guidelines, the present indication for initial PTx for SHPT can be applied for the indication for re-PTx. Localization of causative PTGs can be roughly predicted using the Casanova test, modified Casanova test, or intact PTH ratio. For a detailed investigation, US and MRI are useful for causative PTGs in the forearm; CT and MIBI scintigraphy, or SPECT/CT imaging are useful for causative PTGs in the neck or mediastinum.

### 8.2 Re-PTx for persistent or recurrent SHPT

The indication for re-PTx for persistent or recurrent SHPT is the same as that for initial PTx (34, 35). Two kinds of operations are expected: re-PTx in the neck or mediastinum and re-PTx in the autografted forearm. Re-PTx in the neck can be performed from the same incision as the initial PTx. However, adhesion due to the initial PTx potentiates the risk of RLN injuries and postoperative hemorrhage (56). To prevent RLN injuries, precise preoperative localization of causative PTGs is essential. The usefulness of IONM was demonstrated in patients with re-operation for thyroid tumors (40). However, to the best of our knowledge, there are no studies evaluating the usefulness of IONM for re-PTx. Adhesion of PTGs to RLNs might occur after initial PTx, making it difficult to identify the RLNs. These situations can often be identified during re-PTx, and IONM should be applied for re-PTx cases. Re-PTx for cases of causative PTGs in the mediastinum requires sternotomy or thoracoscopic surgery. These procedures should be performed by experienced endocrine or thoracic surgeons. In addition, IOIPTH monitoring may be useful to confirm the removal of the causative PTGs; however, this has not been investigated to date.

PTG autografting involves the grafting of small, fragmented pieces of PTG under the skin or in the muscle. Re-PTx in the autografted forearm can be performed under local anesthesia. However, persistent SHPT can occur after re-PTx. To prevent persistent SHPT, the autografted PTGs need to be removed to the greatest extent possible. PTGs autografted in the muscle should be removed with the surrounding muscles to prevent very small remnant PTGs. Even after the removal of autografted PTGs, hypoparathyroidism rarely occurs (104). However, investigations on autograft after PTx such as operative methods, risk factors for recurrent SHPT, and repeated recurrent SHPT are still needed (74).

Studies describing the results after re-PTx are limited. Serum calcium levels have been demonstrated to decrease markedly from 10.2 mg/dL to 8.9 mg/dL after re-PTx, although the postoperative serum phosphorus levels were similar to preoperative levels (33). The patient survival rate after re-PTx has not been investigated. However, previous studies have demonstrated an increased mortality in ESRD patients with serum intact PTH levels > 400–600 pg/mL, implying that the re-PTx may improve mortality (118–122).

## 9 Outcomes of calcimimetics treatment and PTx

### 9.1 Cinacalcet hydrochloride

Cinacalcet was the first commercially available calcimimetic. Cinacalcet suppresses PTH production by allosterically attaching to the calcium-sensing receptors on PTGs (123). The MBD-5D study in Japan demonstrated a significant decrease in serum PTH and a favorable control of serum calcium and phosphorus by using cinacalcet with a reduced dose of a vitamin D agent (124). The direct effect of cinacalcet on PTGs has been demonstrated. Cinacalcet may cause histological changes and reduce the volume of enlarged PTGs (125, 126). The effect of cinacalcet on the bone was histologically demonstrated in the BONAFIDE study. In patients with high-turnover bone disease, long-term cinacalcet administration could improve the bone formation rate, bone metabolism markers, and histological findings (127). In the EVOLVE trial, cinacalcet treatment could significantly decrease the incidence of clinical fractures as a secondary endpoint in the prespecified lag-censoring analysis compared with placebo, although statistical significance was not demonstrated in the intention-to-treat analysis (128). Additionally, improvements in mortality and cardiovascular events as the primary endpoint were demonstrated in the cinacalcet treatment group by the prespecified lag-censoring analysis compared with a placebo group, although a significant difference was not demonstrated in the intention-to-treat analysis (129). In the sub-analysis of the EVOLVE trial, the incidence of non-arteriosclerotic cardiovascular events such as sudden death and heart failure significantly decreased in the cinacalcet treatment group, although the incidence of arteriosclerotic cardiovascular events was not significantly different between the groups (130). In contrast, in the ADVANCE study, cardiovascular calcification was significantly prevented in the group receiving low-dose vitamin D sterols and cinacalcet compared with the low-dose vitamin D sterols treatment group (131). In studies in Japan and Australia, cinacalcet treatment improved all-cause mortality and mortality due to cardiovascular events (132, 133). Additionally, anemia was improved by cinacalcet treatment (134). While these beneficial effects were demonstrated, frequent gastrointestinal adverse events such as nausea and vomiting were also reported. Moreover, the probability of the ineffectiveness of cinacalcet due to non-adherence to the medication regimen has been discussed (129, 131, 135).

### 9.2 Etelcalcetide hydrochloride

Following the development of cinacalcet, etelcalcetide hydrochloride, which can be administered intravenously, was developed. In a randomized double-blind double-dummy clinical trial performed in the United States, Canada, European countries, Russia, and New Zealand, the effectiveness of etelcalcetide hydrochloride compared with cinacalcet was investigated. Etelcalcetide hydrochloride and cinacalcet similarly resulted in a >30% decrease in the serum PTH level; however, etelcalcetide hydrochloride was superior in achieving a >50% decrease in serum PTH (136). Etelcalcetide hydrochloride was also able to decrease FGF-23 more effectively than cinacalcet (137). In patients who were refractory to cinacalcet treatment due to poor compliance, SHPT was successfully managed with etelcalcetide hydrochloride (138). However, the frequency of gastrointestinal adverse events caused by etelcalcetide hydrochloride was almost equal to that of cinacalcet (137).

### 9.3 Evocalcet

The third calcimimetic is evocalcet, which can be administered orally. Evocalcet was developed to reduce the gastrointestinal adverse events associated with cinacalcet and etelcalcetide hydrochloride. In a head-to-head analysis, the completion rate to control intact PTH levels within the target range with evocalcet treatment was not inferior to that with cinacalcet. Additionally, the frequency of gastrointestinal adverse events was significantly lower at 18.6% in the evocalcet group compared with 32.8% in the cinacalcet group (139). Further, the effect of evocalcet on the improvement of FGF-23, serum-adjusted calcium, phosphorus, and bone turnover marker levels was similar to that of cinacalcet (139). Additional studies on the clinical outcomes of evocalcet are expected. The safety and efficacy after conversion from cinacalcet to evocalcet and after the long-term administration of evocalcet are expected to be demonstrated in the future (140).

### 9.4 PTx

The most radical and effective treatment for SHPT is PTx. Recently, SHPT refractory to medical treatment has been treated with PTx as a final treatment option. PTH levels dramatically improve after PTx, although calcium and vitamin D administration is indispensable owing to hypocalcemia. The improvement in bone density and the low frequency of bone fracture after PTx were demonstrated in an analysis of data from the United States Renal Data System (9, 141). A propensity score matching analysis of all-cause mortality and cardiovascular-related mortality demonstrated a 34% and 41% additional decrease in the PTx group compared with the non-PTx group for patients with severe SHPT, respectively (7). Similarly, in the PTx group, the all-cause mortality was superior to that in the non-PTx group according to the propensity score matching analysis of the United States Renal Data System data (8). Additionally, the effects of PTx in improving anemia, insomnia, cognitive status, peripheral arterial disease, and blood pressure have been demonstrated (142–146). The cost-effectiveness of PTx compared with medical treatment in patients eligible for PTx was also demonstrated (2, 37). As for the safety of PTx, the mortality rate within 30 days after PTx was only 2.0–3.1% (8, 147).

### 9.5 Calcimimetics treatment versus PTx

The importance of PTx after the development of calcimimetics needs to be discussed, as calcimimetics have dramatically reduced the need for PTx for SHPT. A direct comparison of the improvement in all-cause mortality between calcimimetics and PTx has not yet been reported. However, in a meta-analysis comparing medical treatment involving calcimimetics and PTx, the all-cause mortality and cardiovascular event-related mortality showed greater improvements in patients treated with PTx (148, 149). Moreover, in a prospective cohort study from Japan, PTx-treated patients were compared with matched cinacalcet-treated patients, and PTx reduced the risk of mortality, most prominently in patients with intact PTH levels ≥500 pg/mL and those with serum calcium levels ≥10 mg/dL (2). A recent study of the United States Renal Data System and Japanese Society for Dialysis Therapy Renal Data Registry also showed superior 5-year and 6-year survival in the PTx-treated group compared with the cinacalcet-treated group (2, 150). Additionally, from the viewpoint of QOL, the superiority of PTx compared with cinacalcet was demonstrated in a systematic review, where the physical component score and mental component score in the 36-item Medical Outcomes Study Short-Form Health Survey were significantly more improved in the PTx-treated patients than in the cinacalcet-treated patients (151). Furthermore, PTx is more cost-effective than cinacalcet treatment (26, 27).

Although the number of PTx for SHPT has dramatically decreased after the various developments of calcimimetics, the advantages of PTx should be re-evaluated. PTx can be indicated for several patients, for example, young patients and patients with good general conditions who need long-term dialysis and medical treatment. Further studies on the indication of medical treatment or PTx should be investigated.

## 10 Discussion

The development of medical treatments including calcimimetics has changed the treatment of SHPT (23, 24). With the demonstration of efficacy, treatment for SHPT has changed from PTx to medical treatment. Nevertheless, patients with SHPT refractory to medical treatment exist (25). For these patients, PTx is the best treatment and should be indicated without delay to improve their QOL and reduce mortality (2, 150, 151). However, as the number of PTx for SHPT has decreased, consequently endocrine surgeons who have sufficient experience in performing PTx for SHPT are also decreasing. The difficulty of PTx for SHPT lies in removing all PTGs to prevent persistent or recurrent SHPT. For this purpose, preoperative imaging diagnosis and other intraoperative procedures are critical. Additionally, the appropriate PTx procedure should be selected according to the patients’ condition and their schedule of kidney transplantation (53). Although there are several reviews on PTx for SHPT, there is no specific focus on the procedures for successful PTx, only on the outcomes after PTx. In this review, we focused on methods to achieve successful PTx based on previously published literature. Young endocrine surgeons might only have limited opportunities to perform PTx for SHPT. It is important to select the appropriate methods and modalities to ensure the success of PTx, and these principles should be handed down to the next generations. For such young endocrine surgeons, the authors hope that this review will be valuable. Considering that currently a limited number of studies have compared the safety and efficacy of calcimimetics and PTx, there is need for future studies to guide surgeons in choosing the optimal treatment approach. Moreover, future studies should investigate new modalities and methods to further improve the outcomes of treatment for SHPT.

## Author contributions

TH drafted the manuscript. YH, KF, MO, NG, YT, SN, YW, and TI revised the manuscript. All authors contributed to the article and approved the submitted version.
